# Microvesicles derived from dermal myofibroblasts modify the integrity of the blood and lymphatic barriers using distinct endocytosis pathways

**DOI:** 10.1002/jex2.151

**Published:** 2024-05-02

**Authors:** Syrine Arif, Megan Richer, Sébastien Larochelle, Véronique J. Moulin

**Affiliations:** ^1^ Centre de recherche en organogénèse expérimentale de l'Université Laval/LOEX Centre de recherche du CHU de Québec‐Université Laval Quebec Canada; ^2^ Department of Surgery, Faculty of Medicine Université Laval Quebec Canada

**Keywords:** blood endothelial cells, endothelial barrier, endothelial cell, extracellular vesicles, lymphatic endothelial cells, microvesicles, myofibroblast, regeneration

## Abstract

Microvesicles (MVs) are a subtype of extracellular vesicles that can transfer biological information from their producer cells to target cells. This communication can in turn affect both normal and pathological processes. Mounting evidence has revealed that dermal wound myofibroblasts (Wmyo) produce MVs, which can transfer biomolecules impacting receptor cells such as human dermal microvascular endothelial cells (HDMECs). While the effects of MVs on HDMECs are generally well described in the literature, little is known about the transport of MVs across the HDMEC barrier, and their potential effect on the barrier integrity remains unknown. Here, we investigated these roles of Wmyo‐derived MVs on two sub‐populations of HDMECs, blood endothelial cells (BECs) and lymphatic endothelial cells (LECs). Using an in vitro model to mimic the endothelial barrier, we showed that MVs crossed the LEC barrier but not the BEC barrier. In addition, we demonstrated that MVs were able to influence the cell‐cell junctions of HDMECs. Specifically, we observed that after internalization via the predominantly caveolin‐dependent pathway, MVs induced the opening of junctions in BECs. Conversely, in LECs, MVs mainly use the macropinocytosis pathway and induce closure of these junctions. Moreover, proteins in the MV membrane were responsible for this effect, but not specifically those belonging to the VEGF family. Finally, we found that once the LEC barrier permeability was reduced by MV stimuli, MVs ceased to cross the barrier. Conversely, when the BEC barrier was rendered permeable following stimulation with MVs, they were subsequently able to cross the barrier via the paracellular pathway. Taken together, these results suggest that the study of Wmyo‐derived MVs offers valuable insights into their interaction with the HDMEC barrier in the context of wound healing. They highlight the potential significance of these MVs in the overall process.

## Graphical abstract

Microvesicles originating from dermal myofibroblasts alter the integrity of the blood and lymphatic barriers via different pathways.

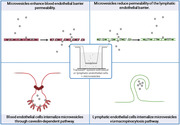

## 1 INTRODUCTION

Over the last decade, the study of extracellular vesicles (EVs) has revealed that they are a crucial mediator in cell‐to‐cell communication (Al Halawani et al., 2022; Kalra et al., 2016; Narauskaitė et al., 2021; Théry et al., 2018; Yuana et al., 2013). Because EVs are detected in the blood, it is assumed that EVs are produced in tissues and then released into the bloodstream to exert an action on other tissues. However, our understanding of the mechanisms of EV transport from tissues to the bloodstream across the endothelial barrier remains very limited.

EVs are small particle entities, consisting of a lipid‐based membrane produced by cells and containing a wide range of biologically active molecules, including proteins and nucleic acids (Théry et al., 2018). EVs play a crucial role in the regulation of various physiological processes, and especially during the different stages of wound healing (Arif et al., 2020; Dalli et al., 2013; Kerris et al., 2020; Lopez et al., 2019; Merjaneh et al., 2017; Zhou et al., 2020). Skin healing is a complex process involving intercellular communication, regulation of the extracellular matrix and the presence of bioactive molecules such as growth factors (Barrientos et al., 2008; Clark, 1988; Martin, 1997; Narauskaitė et al., 2021; Schultz & Wysocki, 2009; Singer & Clark, 1999). During this process, specialized cells called myofibroblasts (Wmyo) are generated from the differentiation of fibroblasts to promote extracellular matrix remodelling and wound contraction (Arif et al., 2021; Hinz et al., 2007). Our team previously reported that Wmyo produce a subtype of EVs called microvesicles (MVs) (Merjaneh et al., 2017; Moulin et al., 2010) that contain various molecules such as vascular endothelial growth factor (VEGF) that stimulate angiogenic mechanisms (Arif et al., 2020; Moulin et al., 2010).

Although the role of EVs in skin wound healing has been extensively studied (Narauskaitė et al., 2021), their transportation out of the tissue has received relatively little attention. In tissues, networks of blood capillaries and lymphatic capillaries provide oxygen and nutrients while removing waste and excess fluid (Fishel et al., 2003; Knezevic et al., 2017; Sarin, 2010; Takada & Hattori, 1972). These capillaries are composed of either blood endothelial cells (BEC) or lymphatic endothelial cells (LECs). During wound healing, capillaries facilitate the exchange of various elements, including cells, proteins and other biomolecules, between the circulation and surrounding tissue (Dixon, 2010; Sarin, 2010; Velnar & Gradisnik, 2018). The efficient exchange of these elements is essential for the normal progression of wound healing. However, the fate of MVs after their released from Wmyo is still unclear. Understanding the behaviour of MVs during diffusion may shed light on their role in skin wound healing and highlight their importance. Therefore, in this study, our objective was to investigate whether and how MVs diffuse across the endothelial barrier.

In this study, we aimed to elucidate the behaviour of MVs derived from Wmyo using a Transwell® system seeded with human dermal endothelial cells (HDMECs). These cells are enriched with either BECs or LECs. Our results revealed that the diffusion of MVs across the barrier was influenced by the origin of the HDMECs used. Additionally, MVs were able to modulate the permeability of the endothelial barrier. Interestingly, we also observed that MVs used different endocytosis pathways depending on the origin of the HDMECs. The results of this study thus suggest that the fate of MVs is influenced by the type of HDMEC. This indicates that MVs play a crucial role in wound healing processes that involve HDMEC functions under physiological conditions.

## 2 MATERIAL AND METHODS

### 2.1 Cell populations and culture

The research protocols were approved by the institutional ethics committee of the CHU de Quebec‐Université Laval Research Center in accordance with the Helsinki Declaration of 1975, as revised in 2008.

Primary myofibroblasts (Wmyo) were isolated from the wounded skin of adult donors aged 20–40 years following a previously described method (Germain et al., 1994). Wmyo were cultured in a medium consisting of Dulbecco's Modified Eagle's medium (DME, 10‐013‐CV, Corning, Manassas, VA) supplemented with 20% fetal bovine serum (FBS, FB essence, Seradigm, Radnor, PA), 100 U/mL penicillin G (Sigma‐Aldrich, Saint‐Louis, MO, USA) and 25 µg/mL gentamicin (Schering Inc., Kenilworth, New Jersey, USA). Cultures were maintained at 37°C in a humidified incubator with 8% CO_2_, and passages 3–8 were used for experimentation.

HDMECs were extracted from skin samples obtained after elective surgery (inner arm, donors of 29 ± 8 years). The epidermis was removed after dispase digestion and HDMECs were isolated from the dermal compartment by extrusion (Bourland et al., 2019; Tremblay et al., 2005). HDMECs in Passage 0 cultures were separated from non‐endothelial cells using immunomagnetic beads coupled to antibodies against CD31 (Dynabeads CD31, 11155D, Invitrogen, AS, Norway). After extraction, HDMECs were seeded in gelatin‐coated flasks and cultured in EGM™‐2 medium (CC‐3156, Lonza, Basel, Switzerland) with additives (CC‐4147, Lonza). This medium is composed of a basal medium supplemented with fetal bovine serum, hydrocortisone, hFGF‐B, VEGF, insulin‐like growth factor, ascorbic acid, epithelial growth factor and gentamicin. Cells were grown at 37°C in a humidified incubator with 8% CO_2_ and were used at passages between 1 and 4.

### 2.2 Production of fluorescent Wmyo

To generate cell populations producing fluorescent MVs, three different populations of Wmyo were transduced with a gene encoding mNeonGreen‐UtrCH (a gift from Dorus Gadella, Addgene plasmid #98879; http://n2t.net/addgene:98879; RRID: Addgene_98879) (Chertkova et al., 2017) as previously described (Mayrand et al., 2012; Merjaneh et al., 2017).

### 2.3 Isolation of MVs

**FIGURE 1:**
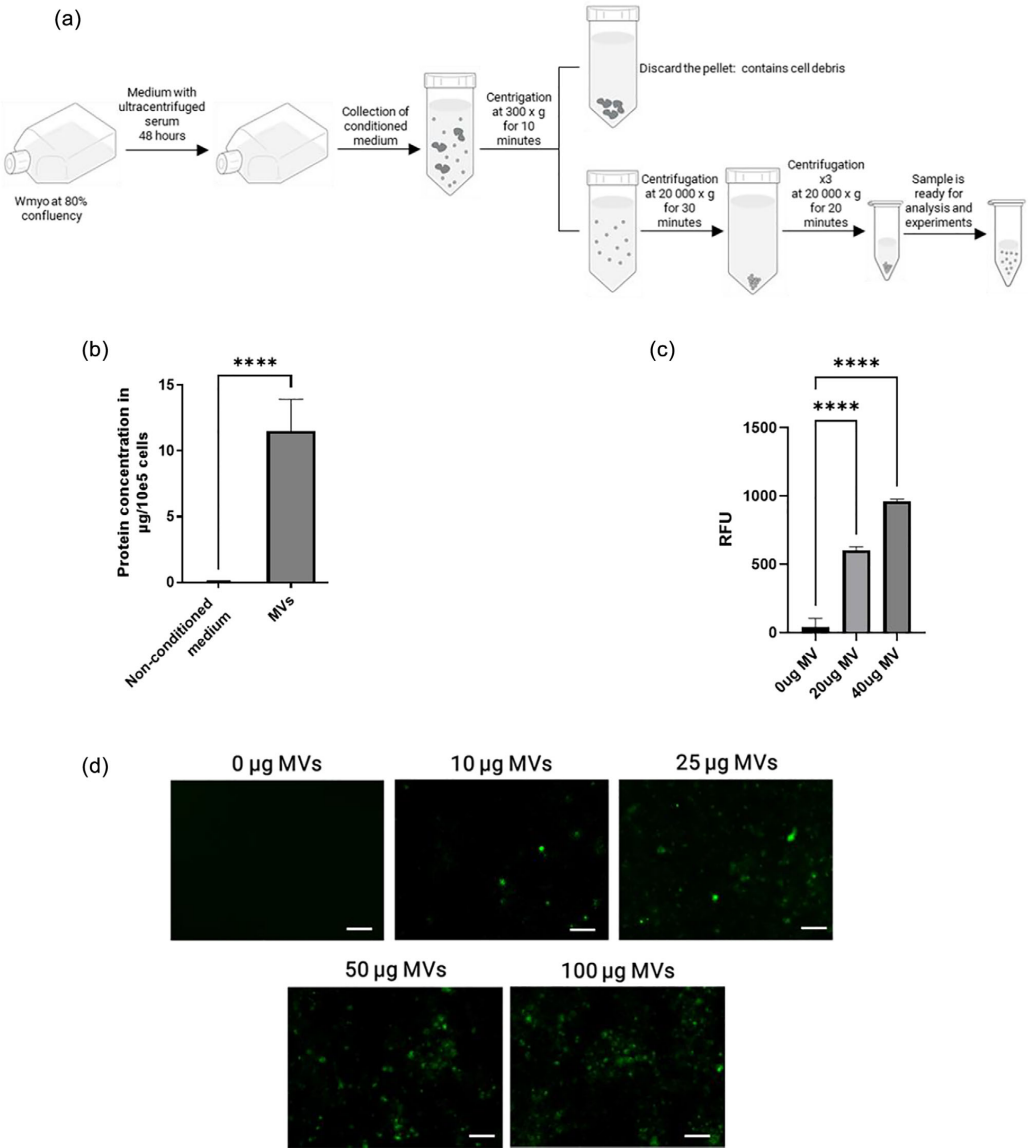
Fluorescent Wmyo produce fluorescent MVs. (a) Schematic diagram of the isolation process of Wmyo‐derived MVs by differential centrifugation. (b) Amount of protein in MVs, produced by one million Wmyo for 48 h and measured using a NanoDrop 1000 spectrophotometer at 280 nm. *N* = 3 MV samples from 3 different Wmyo populations, *n* = 3. An unpaired *T*‐test was used, ****p < 0.0001. (c) Relative fluorescence units (RFU) measured for fluorescent mNeonGreen MVs at 0, 20, and 40 µg of MV protein per well. Results are expressed as the mean ± SD of triplicate experiments. One‐way ANOVA (nonparametric) with Dunnett's test (control: 0 µg of MV protein), ****p < 0.0001. *N* = 3 MV samples from three different Wmyo populations, *n* = 3. (d) Representative photographs of fluorescent mNeonGreen MVs. MV protein amounts were 0, 10, 25, 50, and 100 µg of MV protein per well. The scale bar represents 10 µm. Representative image of *N* = 3 MV samples from three different Wmyo populations, *n* = 3.

MVs were isolated by differential centrifugation as previously described (Arif et al., 2020; Merjaneh et al., 2017) (Figure 1a). Briefly, when Wmyo reached 80% confluence, the culture medium was replaced with DME supplemented with 20% vesicle‐free FBS and antibiotics. After 48 h, the conditioned medium was collected and centrifuged for 10 min at 300 × *g* at 4°C. The resulting supernatant was further centrifuged at 21,000 × *g* for 30 min at 4°C. Lastly, the resulting pellet was washed three times with phosphate buffer saline and subjected to a final centrifugation at 21,000 × *g* for 20 min before being used in the experiments. It is acknowledged that this method may co‐isolate protein aggregates or possible membrane fragments/cell debris. However, in our samples contaminants were in very low concentrations (Arif et al., 2024).

The protein concentration of MV samples was quantified using a NanoDrop 1000 spectrophotometer (Thermo Fisher Scientific, Mississauga, ON, Canada) by measuring absorbance at 280 nm.

### 2.4 Fluorometric assay

HDMECs were grown on 96‐well plate Transwell® culture inserts (HTS Transwell‐96 well plate, Corning, 3385, Kennebunk, ME, USA) with a pore size of 3 µm until a confluent monolayer was formed.

To investigate the diffusion of MVs across the endothelial barrier, cells were treated in the upper chamber with 40 µg per well of MV protein diluted in EGM™‐2 medium containing only 1% serum (total volume for the upper compartment was 75 µL). The lower chamber of the Transwell^®^ plate was filled with 235 µL of EGM™‐2 medium containing only 1% serum. Ten microlitres of conditioned medium from the lower chamber, where MVs were expected to diffuse, was collected at 0, 3, 6, 24, and 30 h, with the same volume replaced by fresh medium each time. The conditioned medium was then added to a 96‐well plate and diluted with 100 µL of distilled water. The plate was analyzed for mNeonGreen fluorescence using a fluorescent plate reader (Varioskan Flash, ThermoFisher Scientific) by measuring the fluorescence intensity at an excitation wavelength (Exλ) of 506 nm and an emission wavelength (Emλ) of 517 nm. In order to establish a baseline and account for any potential autofluorescence or system‐specific artefacts, a no‐seed control well, containing neither cells nor the specific experimental components, was consistently included and its signal subtracted from the experimental wells in subsequent analyses. The relative fluorescence intensity was then normalized to the initial concentration (C/C0; C0 represents the amount of fluorescence at *t* = 0, and C represents the amount of fluorescence at a given time).

### 2.5 Permeability assay

The permeability of the HDMEC monolayer was detected by the horseradish peroxidase (HRP) diffusion assay, as previously described (Chen & Yeh, 2017). Cells were seeded in Transwell® inserts and, at confluence, incubated with 40 µg of MV protein per well, 100 ng/mL recombinant human VEGF (293‐VE, R&D Systems, Minneapolis, MN) or without treatment. Streptavidin‐HRP (DY998, R&D Systems, Minneapolis, MN) was added to a final concentration of 15 µL/mL, a concentration supported by established protocols (Arakawa et al., 2012; Chen & Yeh, 2017; Jaykumar et al., 2022). Cell‐free wells were included as controls for expected total HRP diffusion. Samples from the lower chamber of the Transwell® plates were collected at 3, 6, 24, and 30 h and replaced with medium to maintain constant medium levels. The collected samples were then diluted by adding 100 µL of distilled water and 50 µL of TMB (3,3′,5,5′‐Tetramethylbenzidine, 34028, Thermo Scientific, Rockford, IL) before being incubated for 10 min. The reaction was stopped by adding 25 µL of a stop solution (2N H_2_SO_4_, Sigma Aldrich, 258105, Oakville, ON, Canada). Optical density was measured at 450 nm using a plate reader (Varioskan Flash, Thermo Fisher Scientific, Rockford, IL). To establish a baseline and account for potential experimental artefacts, a no‐seed control well, devoid of cells, HRP and MVs, was consistently included. The signal from this control well was subtracted from the experimental wells during data analysis.

### 2.6 Immunocytochemistry

HDMEC were washed with phosphate‐buffered saline (PBS), fixed in PBS containing 1% paraformaldehyde for 15 min at room temperature, and permeabilized with PBS containing 0.1% Triton X‐100 (Triton X‐100, 601171, Cayman Chemical, Ann Arbor, MI) for 10 min and blocked with 1% bovine serum albumin (69760, Proliant, New Zealand) for 1 h. The cells were then incubated overnight at 4°C with primary antibodies (see Table 1: CD31; Podoplanin; LYVE‐1; PROX‐1; Ve‐Cadherin; ZO‐1; isotypes). Cells were then washed with PBS and incubated with appropriate secondary antibodies (see Table 1: Alexa 546 goat anti‐mouse IgG1 or Alexa 594 goat anti‐rabbit IgG) for 1 h at room temperature. Hoechst (33258, Sigma, Oakville, ON, Canada) was used to stain cell nuclei at a 1:100 dilution for 10 min at room temperature. The cells were visualized by fluorescent microscopy using an Axio Imager.M2 microscope (Zeiss, Toronto, ON, Canada) equipped with an Axiocam ICc1 camera (Zeiss) and Zen software (Zeiss).

**TABLE 1:** Primary and secondary antibodies used for immunolabelling.

Antibody	Host	Type	Company	Catalogue number	Dilution
CD31	Mouse	IgG1	BD pharmigen	555444	1/400
Podoplanin	Mouse	IgG1	Angio‐Bio Co.	11‐003	1/500
LYVE‐1	Rabbit	IgG	Fitzgerald	70R‐LR003	1/100
PROX‐1	Rabbit	IgG	Abcam	Ab37128	1/50
Ve‐Cadherin	Mouse	IgG1	BD pharmigen	555661	1/400
ZO‐1	Rabbit	AcP	ThermoFisher	40‐2200	1/50
Isotype	Mouse	IgG1	Dako	X0931	1/100
Isotype	Rabbit	IgG	R&D Systems	AB‐105‐C	1/100
Hoechst	–	–	Sigma	33258	1/100
Anti‐Mouse Alexa 546	Goat	IgG1	Invitrogen	A21123	1/1000
Anti‐Rabbit Alexa 594	Goat	IgG	Invitrogen	A11012	1/1600

The internalization of fluorescent MVs by endothelial cells was quantified with ImageJ software (https://imagej.nih.gov/ij/) by delineating the CD31‐labelled cell membrane.

The effect of MVs on endothelial cell junctions was assessed using the gap index and gap size index quantified according to the method described by Fraccaroli et al. (2015).

### 2.7 Inhibition of endocytosis

To investigate the effects of various inhibitors of endocytosis on MV incorporation by HDMECs, we seeded cells at 50,000 cells per well in 96‐well plates and allowed them to reach confluence. Cells were then exposed to different concentrations of inhibitors for 30 min. To assess the cytotoxicity of the inhibitors, cell viability was determined using alamarBlue (DAL1025, Invitrogen, Burlington, ONT, Canada) according to the manufacturer's instructions. Inhibitor concentrations that did not show toxicity in BECs and LECs were chosen for the subsequent experiments (Supp. data 1): amiloride (A7410, Sigma‐Aldrich, Oakville, ON, Canada) at 0.5 mM/mL, dynasore (D7693, Sigma‐Aldrich, Oakville, ON, Canada) at 50 µM/mL, nystatin (N1638, Sigma‐Aldrich, Oakville, ON, Canada) at 25 µL/mL, and chlorpromazine (C8138, Sigma‐Aldrich, Oakville, ON, Canada) at 5 µM/mL. Inhibitors were added to HDMECs, and the cells were incubated at 37°C for 30 min before the addition of MVs and the evaluation of permeability.

To evaluate the effects of endocytosis inhibitors on MV internalization, cells were fixed, permeabilized and stained with VE‐cadherin antibodies. The relative fluorescence intensity of mNeonGreen (RFI)/cell was quantified by analyzing five randomly selected images per condition using ImageJ.

### 2.8 Digestion of MV membrane proteins

The extravesicular domains of MV membrane proteins were digested with 20 µg/mL proteinase K (PK) (PB0451, BioBasic, Canada) according to the method described by Skliar et al. (2018). The mixture was incubated at 37°C for 1 h, after which the PK activity was terminated by heating the sample to 65°C for 10 min. To remove the remaining debris, MVs were centrifuged at 20,000 × *g* for 20 min. Subsequently, the PK‐treated MVs were used for the permeability assay.

### 2.9 Neutralization of VEGF receptors on HDMECs

To investigate the effect of Vascular Endothelial Growth Factor Receptor (VEGFR) signalling on endothelial barrier permeability, the cells were grown to confluence in Transwell® inserts and treated with 15 µg/mL of hVEGFR1 (Flt‐1) antibody (anti‐hVEGFR1 (Flt‐1), AF321, R&D, Minneapolis, MN, USA), 0.5 µg/mL of hVEGFR2 antibody (anti‐hVEGFR2, MAB3572, R&D) or 5 µg/mL of VEGFR3 antibody (anti‐VEGFR3, MAB3757, Millipore, Oakville, ON, Canada) diluted in EGM™‐2 containing 1% serum for 1 h. After incubation, HDMECs were washed with EGM™‐2, and the treatments were added. The medium was sampled after 3 h and analyzed for permeability as described previously.

### 2.10 Statistical analysis

All statistical analyses were performed using GraphPad Prism software (GraphPad Prism 9.4.0, San Diego, CA). Results were reported as mean ± standard deviation (SD), unless otherwise stated. All experiments were performed with three different cell populations to ensure biological reproducibility. Statistical significance was defined as *p* < 0.05, with methods detailed in the figure legends.

## 3 RESULTS

### 3.1 Characterization of fluorescent myofibroblast‐derived microvesicles

Fluorescent MVs were obtained after the transduction of Wmyo cells with the mNeonGreen gene, as described in the methods section. MVs produced by Wmyo have been extensively characterized in our previous studies (Arif et al., 2020; Merjaneh et al., 2017; Moulin et al., 2010). In addition to the routine evaluation of MVs by Nanodrop analysis, we also quantified their presence using fluorescence methods.

MVs were first isolated by differential centrifugation from the conditioned media of Wmyo cultures (Figure 1a). MVs had a protein concentration of 11.51 ± 2.28 µg of protein/10^5^ Wmyo cells (Figure 1b). The presence of fluorescent MVs was confirmed using a plate reader, which showed an increase in relative fluorescence units with the increasing concentration of MVs in the wells (Figure 1c). Fluorescent microscopy was also used to detect the presence of MVs (Figure 1d). An increase in fluorescent MVs was observed with increasing MV concentration. However, as the detection of MVs is limited by the resolution of the microscopy, the observed fluorescence represents the fluorescence emission produced by the fluorophore, rather than the individual vesicles themselves, as has been noted in other studies (Abbaszade Dibavar et al., 2018; Campello et al., 2022; Dehghani et al., 2020; Ter‐Ovanesyan et al., 2017). Our results indicate that fluorescent Wmyo produced fluorescent MVs that can be detected by fluorescence methods without the need for exogenous markers.

In addition to the above‐mentioned features, extensive characterization of MVs have been conducted (Arif et al., 2020, 2024; Merjaneh et al., 2017; Moulin et al., 2010), adhering to the MISEV guidelines. Our studies have shown that MVs display a weak expression of exosomal markers, such as CD81 or CD63, while exhibiting a strong positive labelling for Annexin V (Arif et al., 2024; Moulin et al., 2010). Morphologically, isolated MVs present a well‐defined spherical shape encapsulating a discernible lipid bilayer (Arif et al., 2020, 2024). The size distribution of MVs, as determined by both transmission electron microscopy analysis and cryo‐electron microscopy analysis, spans approximately 116 and 176 nm, respectively (Arif et al., 2020, 2024). Moreover, prior investigations have definitively established the absence of apoptotic bodies within MVs derived from dermal Wmyo (Moulin et al., 2010). These comprehensive characterizations collectively reinforce the unequivocal identification of MVs as a distinct subtype within the realm of extracellular vesicles.

### 3.2 Characterization of blood and lymphatic endothelial cell subpopulations enriched from human dermal microvascular endothelial cell cultures

HDMECs were extracted from the dermis of adult skin using a method based on enzymatic digestion and mechanical extrusion, as described in a previous study (Bourland et al., 2019). HDMECs are composed of two different subpopulations of cells, namely BEC and LEC.

**FIGURE 2:**
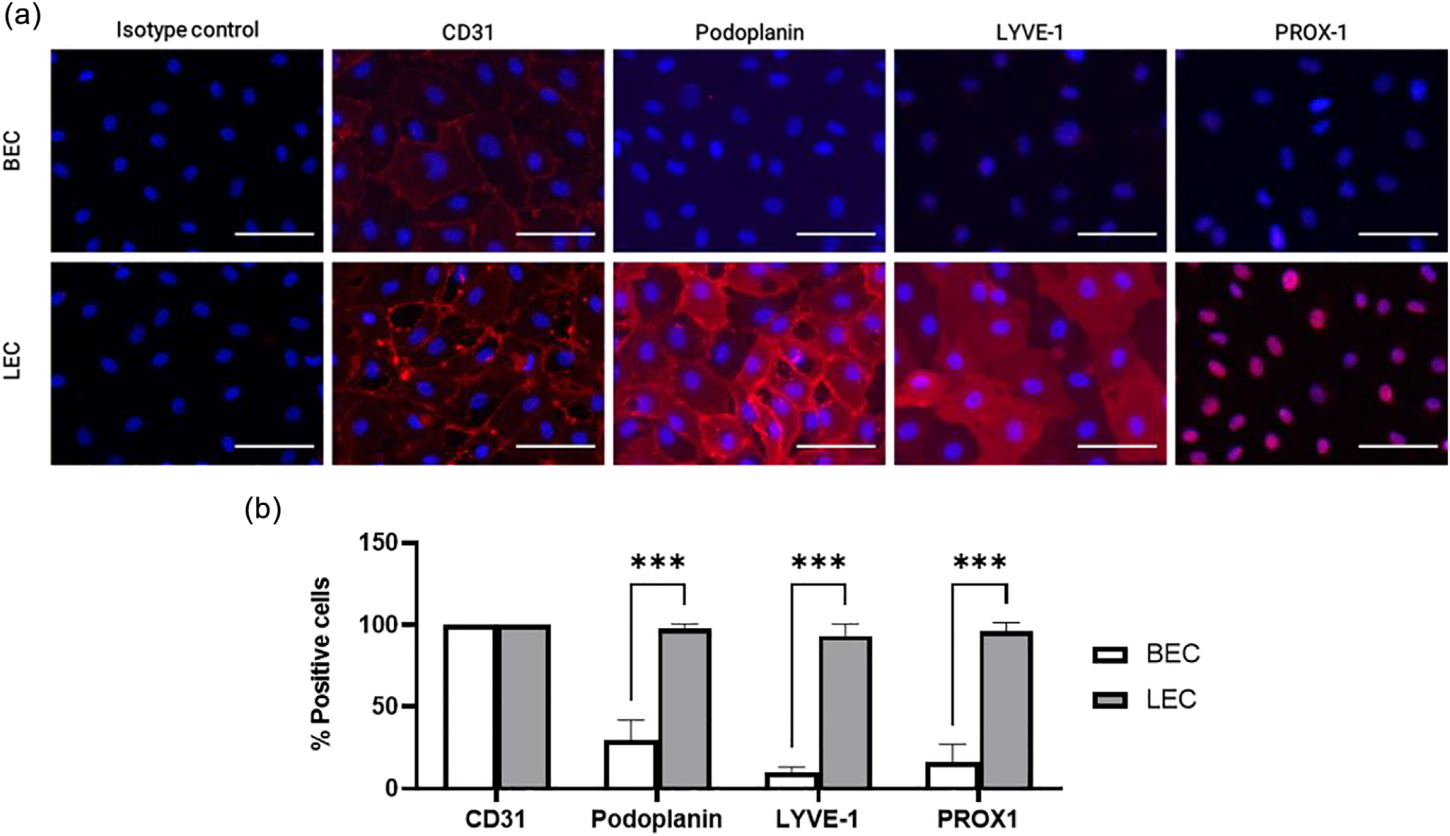
Characterization of BEC‐ and LEC‐enriched HDMEC cultures. Isolated HDMECs were characterized using immunofluorescence labelling. (a) Immunofluorescence staining for endothelial markers is depicted for each type of enriched HDMEC population. Blue represents the Hoechst counterstaining of the nuclei and red represents positive cells for the indicated markers (CD31, podoplanin, LYVE‐1 and PROX‐1). The scale bar represents 100 µm. Photos are representative of a cell population for *N* = 3, *n* = 2–3. (b) The percentage of positively labelled cells was analyzed over five images for each of the three subpopulations. A multiple *T*‐test with the two‐stage step‐up method of Benjamini, Krieger and Yekutieli was used, ***p <  0.001. *N* = 3 for each HDMEC population, *n* = 3.

To characterize HDMEC subpopulations, we performed immunofluorescence experiments using specific markers. We selected CD31 as a marker that detects both LEC and BEC subtypes (Kriehuber et al., 2001), but also used podoplanin (Knezevic et al., 2017; Kriehuber et al., 2001), LYVE‐1 (Banerji et al., 1999; Knezevic et al., 2017) and PROX‐1 (Norgall et al., 2007) as markers primarily expressed by LECs. Our observations revealed that a low percentage of BEC‐enriched populations exhibited LEC markers, such as podoplanin (29.4 ± 10.21%), LYVE‐1 (9.6 ± 2.79%) and PROX‐1 (15.93 ± 9.12%) (Figure 2a,b). Conversely, LEC‐enriched populations showed a much higher percentage of cells positive for podoplanin (97.67 ± 2.46%), LYVE‐1 (93.27 ± 5.92%) and PROX‐1 (95.73 ± 4.63%) (Figure 2a,b).

Our findings indicate that two subpopulations are present in the HDMEC samples obtained by the same extraction method. The only variation was observed in the level of enrichment of LECs or BECs in the cell populations. No correlation was observed between the proportion of the HDMEC subpopulations and the patient age or gender (Data not shown). On this basis, we classified cells that had a higher proportion of LEC markers (Podoplanin, LYVE‐1 and PROX‐1) as ‘LEC’, whereas the remaining cells were classified as ‘BEC’. Before performing any experiments in this study, the cell subpopulations were checked in parallel with all other experiments.

### 3.3 MVs crossed the LEC barrier but not the BEC barrier

**FIGURE 3:**
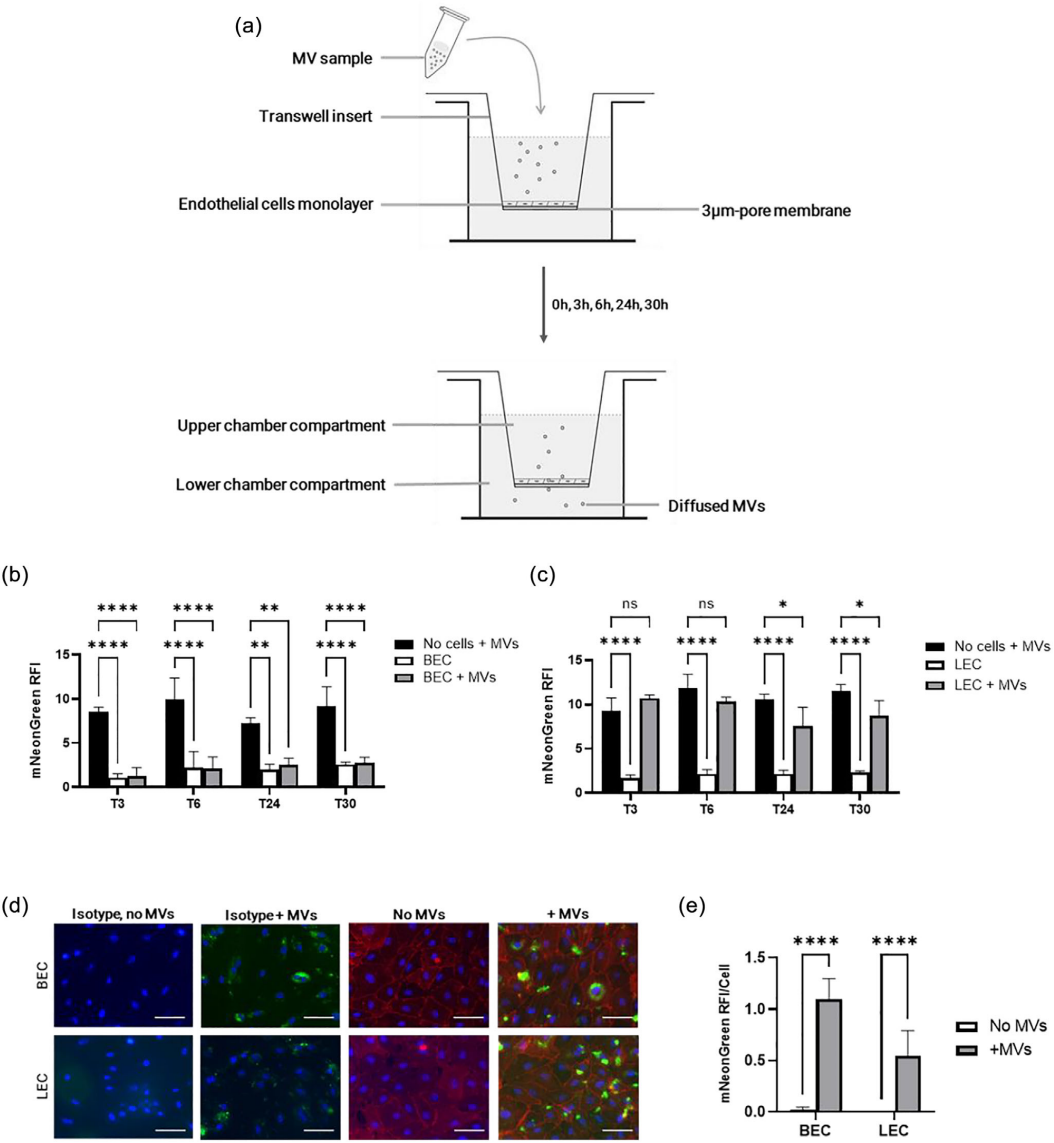
MV diffusion through the endothelial barrier. (a) Schematic representation of the endothelial barrier model used in the study. A measure of the relative fluorescence intensity was used to assess the extent of diffusion of MVs across a BEC barrier (b) and an LEC barrier (c) into the lower transwell compartment. *N* = 3, *n* = 3, *n*’ = 3 MV samples isolated from three different Wmyo populations. The statistical test used was two‐way ANOVA with Dunnett's test (control: No cells + MVs), *p < 0.05, **p <  0.01, ****p <  0.0001. (d) Immunofluorescence microscopy was used to visualize the presence of mNeonGreen‐labelled MVs in BEC and LEC 3 h after stimulation. A representative image of *N* = 2, *n* = 2 is shown, where red indicates CD31 labelling of the cell membrane, blue indicates Hoechst staining of the nuclei, and green indicates the presence of mNeonGreen‐labelled MVs. The scale bar corresponds to 50 µm. (e) Quantitative immunofluorescence analysis shows the relative fluorescence intensity per cell for four images for each duplicate. A multiple *T*‐test with two‐stage step‐up method of Benjamini, Krieger and Yekutieli was used, ****p <  0.0001.

We set up an in vitro transwell assay to determine whether MVs can cross the endothelial barrier (Figure 3a). HDMECs were cultured on the inserts of a 96‐well transwell plate containing a permeable membrane. Forty micrograms of EV protein per well of fluorescent mNeonGreen‐MVs were added to the upper compartment of the inserts containing a confluent layer of HDMEC. Theoretically, if MVs crossed the endothelial barrier, they would be detectable in the lower chamber. Conditioned medium from the lower chambers was collected at different time points (3, 6, 24, and 30 h). The presence of fluorescent mNeonGreen‐MVs was detected by fluorometric assay using a plate reader.

The result showed that MVs did not pass through monolayers of BEC‐enriched HDMECs, even after 30 h (Figure 3b). This suggests that the physiological barrier formed by BECs is not permeable to MVs. In contrast, MVs were able to cross the LEC‐enriched HDMEC barrier within 3–6 h (Figure 3c).

The conditioned medium from the upper chambers was also collected at the end of the experiment, that is, after 30 h. Unexpectedly, we did not observe the presence of fluorescent MVs in the media regardless of the HDMEC subtypes used (Supp. Data 2). These results implied that the MVs were largely internalized by cells. To verify this, we performed an immunofluorescence analysis. Our results revealed that BECs and LECs internalized mNeonGreen‐MVs (Figure 3d,e). The relative fluorescence intensity (RFI) of mNeonGreen per cell was 0.02 ± 0.02 in untreated BECs, compared with 1.09 ± 0.19 for MV‐treated BECs. Similarly, the RFI/cell was 0.008 ± 0.001 in untreated LECs, compared with 0.55 ± 0.23 in MV‐treated LECs. These findings indicate that both types of enriched HDMECs have the ability to internalize MVs. It was also observed that the MVs were mainly localized in the cytoplasmic region and near the nucleus of the endothelial cells (Supp. Data 3).

Evidence of MV internalization by HDMECs prompted us to investigate whether MV internalization had an effect on the endothelial barrier integrity. We therefore explored the potential effects of these vesicles on barrier permeability, which is a crucial factor in wound healing.

### 3.4 MV uptake increased the permeability of the BEC barrier but decreased the permeability of the LEC barrier

**FIGURE 4:**
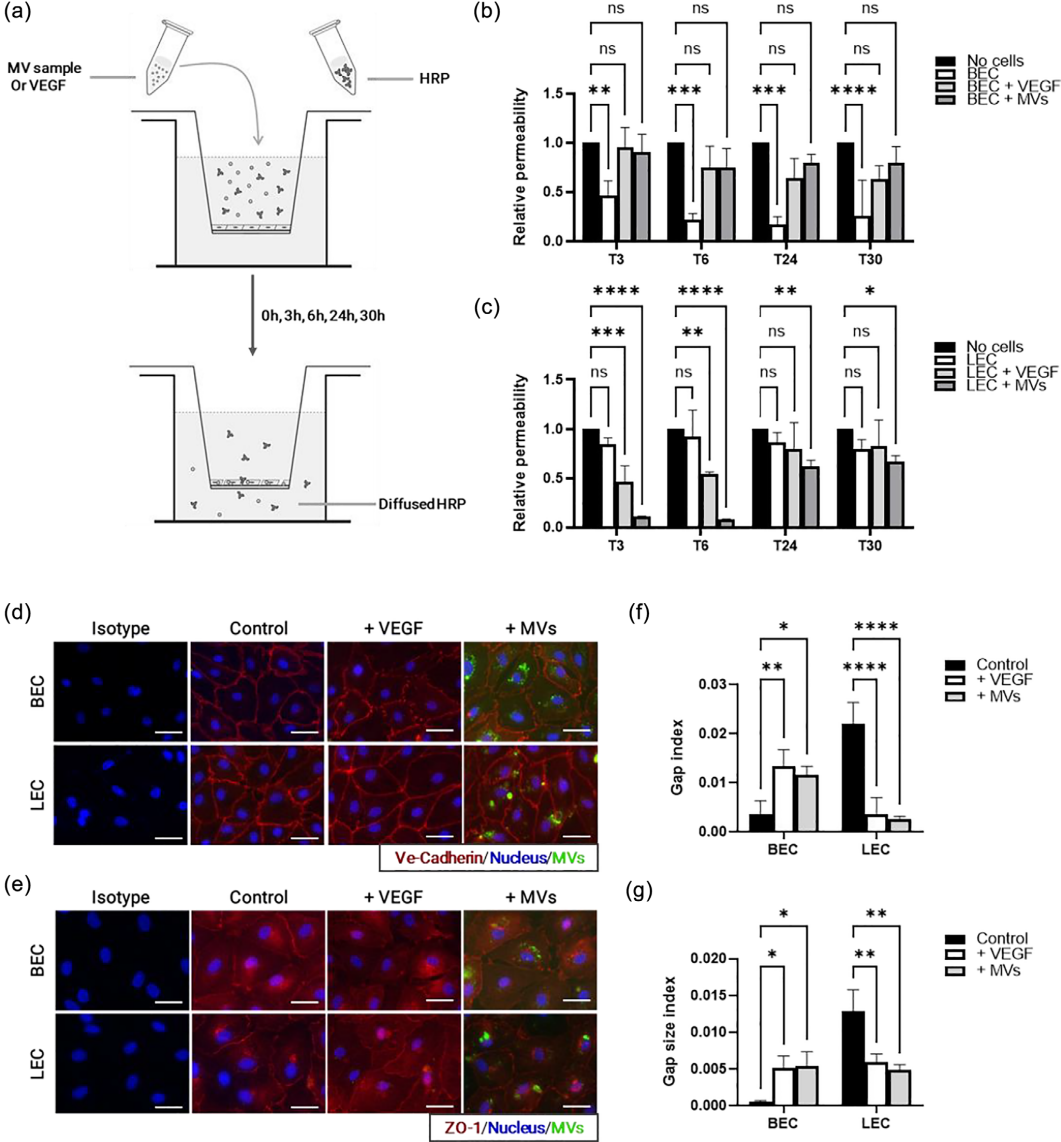
Effect of MVs on barrier integrity. (a) Schematic illustration of the experimental workflow. After seeding on transwell inserts and reaching confluence, the HDMEC monolayer was treated with 40 µg MV protein per well, 100 ng/mL VEGF or no treatment. HRP was added to the treatments in parallel. Samples were collected at 3, 6, 24, and 30 h from the lower transwell chamber. Cell‐free control wells were included, which were treated with HRP only. After detection of HRP by a colorimetric method, the quantitative results of barrier permeability for BEC (b) and LEC (c) were assessed. Data represent means ± SD of the relative permeability for *N* = 3 populations of each HDMEC in triplicate and *n*’ = 3 MVs from three different donors. Statistical analysis was performed using two‐way ANOVA with Dunnett's multiple comparison test. Significance levels are indicated as *p < 0.05, **p < 0.01, ***p < 0.001, ****p < 0.0001, ns *p* > 0.05 compared with the respective control (HRP with no cells in the transwell: ‘No cells’). (d–g) Immunofluorescence staining was performed to assess inter‐endothelial junctions. Cell–cell junctions were labelled with Ve‐cadherin (red) (d) or ZO‐1 (red) (e), while nuclei were stained with DAPI (blue), and MVs were labelled with mNeonGreen (green). The scale bar corresponds to 50 µm. Quantitative analysis of the inter‐endothelial gap index (f) and the gap size index (g) was performed on *N* = 3 HDMEC sub‐populations in triplicate and *n*’ = 1–3 MVs from 1 to 3 different donors. Statistical analysis was performed using two‐way ANOVA with Dunnett's multiple comparison test. Significance levels are indicated as *p < 0.05, **p < 0.01, ***p < 0.001, ****p < 0.0001, ns: *p* > 0.05, compared with the respective control (untreated cells: ‘Control’).

To assess the potential effect of MVs on HDMEC barrier permeability, we used the same transwell model as before but with the addition of HRP as a tracer molecule (Figure 4a). With a hydrodynamic size of approximately 100 nm (Tan et al., 2016), HRP is a stable 44 kDa small molecule that is commonly used to assess endothelial barrier permeability due to its reliability and convenience (Chen & Yeh, 2017; Duffy & Murphy, 2001). In this assay, HRP was added to the upper chamber of the transwell system containing the treated HDMEC monolayer. If the HDMEC barrier was permeable, HRP passed through the barrier and into the lower chamber. Conversely, if the barrier was impermeable, the HRP remained in the upper chamber. To detect HRP, a colorimetric assay was used with a substrate that is converted to a coloured product by an enzyme. The intensity of the colour produced is directly proportional to the amount of HRP activity present in the sample.

To assess the potential effects of MVs on endothelial barrier permeability, samples were collected from the lower chambers at different time points following treatment. Treatment with MVs resulted in a significant increase in the amount of HRP in the lower compartment when BECs were used, starting 3 h after the treatment (Figure 4b). This indicates an increased permeability of the barrier of BECs in the presence of MVs compared with untreated cells. VEGF treatment was used as a positive control as it is well documented to increase endothelial barrier permeability (Collins et al., 1993; Larcher et al., 1998; Senger et al., 1983). Moreover, later results showed that this factor was detected in MVs (Arif et al., 2020; Moulin et al., 2010). When LECs were used, barrier permeability was high without any treatment, but MV treatment resulted in a significant reduction in barrier permeability at 3 and 6 h (****p < 0.0001). This permeability gradually reversed after 24–30 h (*p < 0.05) and returned to its initial level (Figure 4c). A similar response was observed when VEGF was used, but the effect was less pronounced compared with MV treatment (Figure 4c).

To investigate the action of MVs on HDMEC subtypes, immunofluorescence staining of the tight junction protein Ve‐cadherin and the adherent junction protein ZO‐1 was performed, because the regulation of permeability is closely associated with the status of cell–cell junctions (Anderson & Van Itallie, 1995; Lampugnani et al., 1995; Van Itallie et al., 2009) (Figure 4d,e). After a 3‐h treatment, the cells were fixed and prepared for the experiment. Our results indicate that treatment of BECs with MVs disrupted cell junctions and lead to an increase in intercellular gaps (Figure 4d,e), resulting in an increase in barrier permeability when BECs were used. On the other hand, treatment of LECs with MVs showed a decrease in the presence of intercellular gaps compared with untreated cells (Figure 4d,e). We further quantified these observations using the gap index, which represents the number of intercellular gaps per cell. BECs treated with MVs exhibited a significantly higher gap index than untreated BECs, whereas LECs showed the opposite effect with a reduced gap index following treatment (Figure 4f). In addition, the gap size index, which represents the area of intercellular gap per cell, revealed that gaps in MV‐treated BECs were significantly larger than those in untreated BECs, whereas in LECs, the gap size index decreased after treatment with MVs (Figure 4g).

These findings suggest that MVs differentially affect the permeability of BECs and LECs by altering their cell junctions. Interestingly, MVs and VEGF have comparable effects on HDMECs, indicating that the VEGF component of MVs may be responsible for the observed effect.

### 3.5 The internalization pathway of MVs affected their effect on HDMECs

**FIGURE 5:**
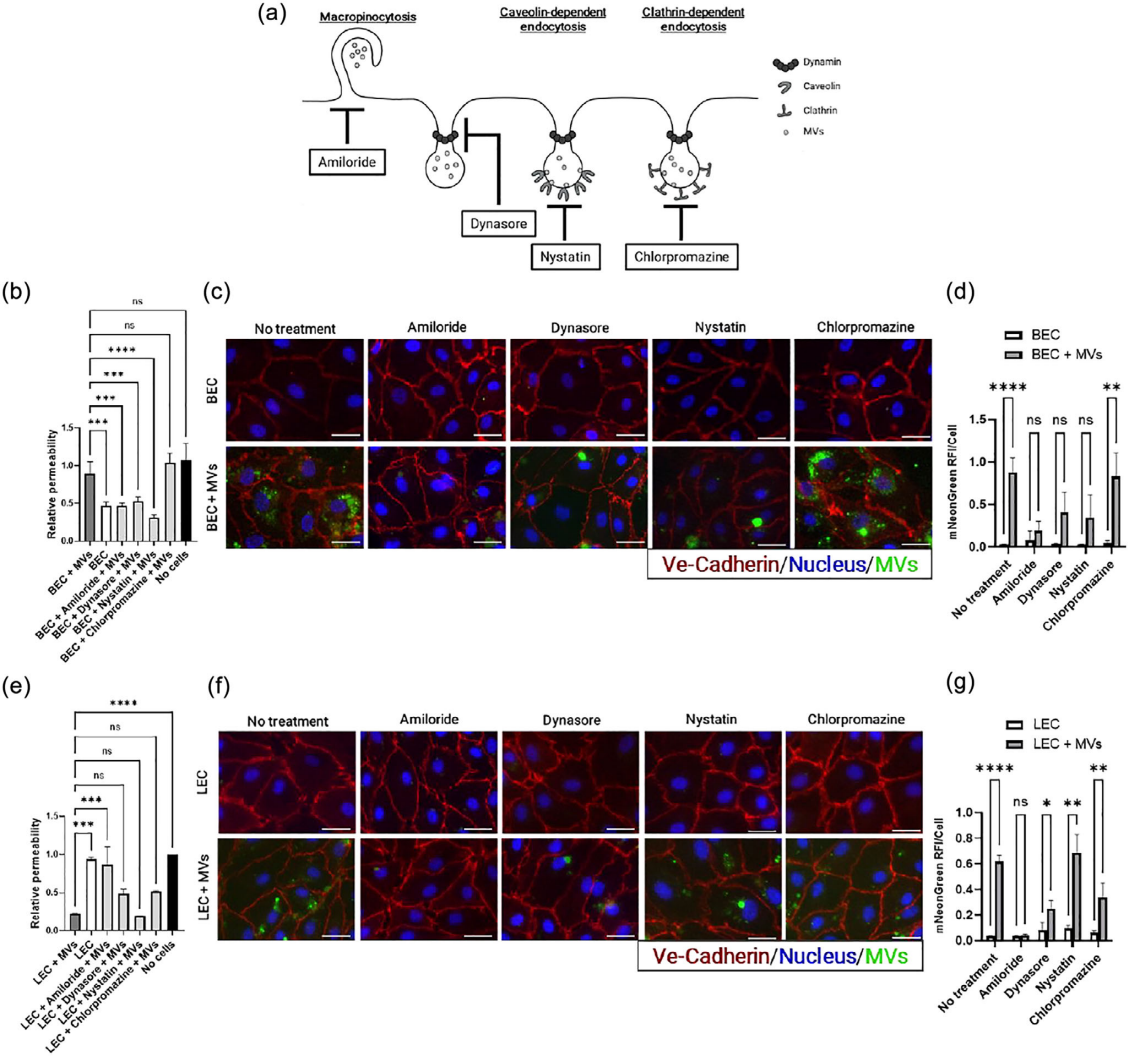
Proposed mechanisms of MV endocytosis by HDMEC subtypes. (a) Schematic representation of the hypothesized mechanisms underlying MV internalization by BECs and LECs as well as the corresponding inhibitors used in this study. The effect of inhibitors on BEC (b–d) and LEC (e–g) barrier permeability was evaluated. (b and e) show the effect of different inhibitors on HRP diffusion, measured by transwell assays. *N* = 2–3, *n* = 3, *n*’ = 1–2. Statistical analysis is presented as mean ± SD of the relative permeability, with one‐way ANOVA followed by Dunnett's test, with ‘HDMEC + MVs’ as the control. ns: *p* > 0.05, **p < 0.01, ***p < 0.001, ****p < 0.0001. (c and f) represent immunofluorescence staining of Ve‐cadherin (in red), nucleus (in blue) and mNeonGreen MVs (in green) with the inhibitors. The scale bar corresponds to 50 µm. (d, g) show quantitative results for fluorescent MVs/cell. *N* = 1–2, *n* = 2–3, *n*’ = 1. Statistical analysis is presented as mean ± SD of relative fluorescence intensity per cell, with multiple unpaired *t*‐tests and the two‐stage step‐up method of Benjamini, Krieger and Yekutieli, with ‘BEC’ or ‘LEC’ as control. ns: *p* > 0.05, *p < 0.05, **p < 0.01, ****p < 0.0001.

To explore the internalization pathways of MVs in HDMEC, we conducted the transwell permeability assay with the assessment of HRP diffusion in the presence of various inhibitors of endocytosis. MVs can be internalized into target cells by different mechanisms such as macropinocytosis, caveolae‐mediated endocytosis, clathrin‐mediated endocytosis, plasma membrane fusion and phagocytosis (Mulcahy et al., 2014) (Figure 5). To investigate the role of these pathways in transcellular trafficking of MVs across the HDMEC monolayer, we employed several inhibitors of endocytic transport, including amiloride to block macropinocytosis by inhibiting Na^+^/H^+^ exchange (Tu et al., 2021); dynasore to inhibit dynamin activity, a GTPase protein involved in endocytosis (Menck et al., 2013; Nanbo et al., 2013); nystatin to inhibit lipid raft/caveolae‐mediated endocytosis (Ginini et al., 2022; Lajoie & Nabi, 2007; Yuan et al., 2017); and chlorpromazine to inhibit clathrin‐dependent endocytosis (Escrevente et al., 2011; Feng et al., 2010) (Figure 5a).

When BECs are used to form the endothelial barrier, the MV‐induced increase in barrier permeability was significantly inhibited by amiloride (***p < 0.001), dynasore (***p < 0.001) and nystatin (****p < 0.0001) but not chlorpromazine (Figure 5b). Immunofluorescence analysis showed that these inhibitors with the exception of chlorpromazine decreased the uptake of MVs by BECs; amiloride (RFI/cell: 0.10 ± 0.09 compared with 0.07 ± 0.19 for the control), dynasore (RFI/cell: 0.004 ± 0.199 compared with 0.04 ± 0.40 for the control), and nystatin (RFI/cell: 0.005 ± 0.23 compared with 0.03 ± 0.35 for the control) (Figure 5c,d). The decrease in MV uptake following these inhibitor treatments suggests that macropinocytosis and caveolae‐dependent endocytosis are likely pathways for MV internalization into BECs.

In contrast, when LECs were used, the MV‐induced decrease in permeability was significantly inhibited only by amiloride (***p < 0.001) (Figure 5e). Immunofluorescence analysis indicated that macropinocytosis endocytosis (RFI/cell: 0.04 ± 0.007 compared with 0.04 ± 0.004 for the control) was largely involved in MV internalization in LECs (Figure 5f,g). The decrease in MV uptake following these inhibitors suggests that macropinocytosis is likely the pathway for internalization of MVs into LECs.

In addition, we also assessed the presence of fluorescent MVs inside the cells for up to 4 days by fluorescence microscopy. We observed that BECs exhibited consistently high levels of fluorescent MVs for up to 4 days. In contrast, LECs showed a decrease in fluorescent MVs after 3 h and fluorescence was almost absent after 4 days (Supp. Data 4). These observations are consistent with the endocytosis pathways determined previously. Indeed, caveolae‐dependent endocytosis, unlike macropinocytosis, avoids the fusion of internalized vesicles with lysosome and vesicle degradation (Kiss, 2012; Kiss & Botos, 2009). Collectively, these results suggest that the mechanisms underlying MV internalization differ between BECs and LECs. In BECs, caveolae‐dependent endocytosis is a primary pathway for MV internalization, whereas in LECs, macropinocytosis plays an important role.

### 3.6 The VEGF family was not responsible for MV internalization

In our previous studies, we identified some members of the VEGF family in MVs such as VEGFA, VEGFD and PlGF (Arif et al., 2020). Since VEGF receptors reside in caveolin (Labrecque et al., 2003) pits and can also be internalized via macropinocytosis (Basagiannis et al., 2016), we investigated whether these factors play a role in MV internalization.

**FIGURE 6:**
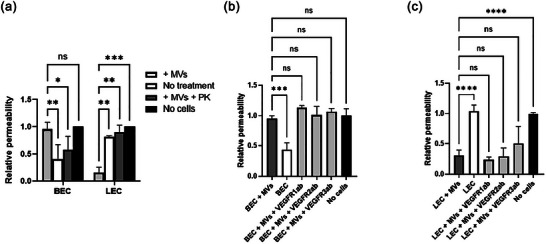
MV membrane proteins and their implication in the barrier permeability. (a) Relative permeability of BEC and LEC in response to treatments with proteinase K‐treated MVs. *N* = 2–3, *n* = 3, *n*’ = 1. Statistical analysis was performed using a two‐way ANOVA, and Dunnett's test with ‘+ MVs’ as the control. Statistical significance is indicated as follows: not significant (ns): *p* > 0.1234; *p < 0.05; **p < 0.01; ***p < 0.001. In addition, neutralization experiments were carried out to assess the involvement of VEGF receptors in MV internalization. Cells were neutralized with antibodies against VEGFR1, VEGFR2 and VEGFR3 for 1 h prior to permeability assays for 3 h. (b and c) Relative permeability of the BEC and LEC barriers, respectively, under various conditions. *N* = 2, *n* = 3, *n*’ = 1. Statistical analysis was performed using one‐way ANOVA and Dunnett's test with ‘HDMEC + MVs’ as control. The statistical significance is indicated as follows: not significant (ns): *p* > 0.1234; ***p < 0.001; ****p < 0.0001.

To address this, we used proteinase K (PK) digestion to cleave the extravesicular domains of proteins on the surface of MVs. PK is a nonspecific protease that digests the majority of surface proteins on EVs (Skliar et al., 2018). We treated MVs with PK and then added them to cells in transwells for permeability assay. Our results indicate that PK treatment of MVs inhibited their effect on permeability for both enriched HDMEC subtypes (Figure 6a). This suggests that MV surface proteins are involved in the MV internalization.

Moreover, as MVs can contain VEGF in their membrane, and VEGF can bind to various receptors (Simons, 2012), we investigated whether the receptors present on endothelial cells are involved in MV internalization. We thus neutralized the cell receptors with antibodies against VEGFR1, VEGFR2 and VEGFR3 for 1 h before conducting the permeability assay for 3 h. Our results show that the neutralization of these receptors did not inhibit the effect of MVs on BEC or LEC permeability (Figure 6b,c). This suggest that VEGF present in the membrane of MVs is not responsible for the effect of MVs on barrier permeability.

## 4 DISCUSSION

The state of the endothelial barrier and fluid exchange between damaged tissue and the circulation are essential for successful wound healing (Braverman, 2000). As EVs have been reported to cross the endothelial barrier and circulate in vessels (Yuana et al., 2013), our aim was to investigate whether MVs produced by Wmyo during cutaneous healing are capable of permeating the endothelial barrier and diffusing into the vascular system.

Despite the presence of different types and locations of endothelial barriers, most studies of EV diffusion across endothelial barriers have mainly focused on the blood‐brain barrier, which has been shown to be either permeable or impermeable to EVs depending on the conditions. To our knowledge, no studies have evaluated the diffusion of EVs across the BEC and LEC barrier and their effect on the permeability of these barriers using cells isolated from capillaries. We first demonstrated that, in the absence of prior endothelial barrier stimulus, MVs did not diffuse across the BEC barrier but diffused freely across the LEC barrier, reflecting the known degree of permeability of blood capillaries as compared with the lymphatic system (Casley‐Smith, 1964; Jannaway & Scallan, 2021; Scallan & Jannaway, 2022; Zhang et al., 2020). We then assessed the action of MVs on barrier permeability as a function of endothelial cell origin.

The BEC barrier is characterized by low permeability to larger molecules, limiting their passage mainly via the paracellular pathway (Egawa et al., 2013; Ono et al., 2017). During skin healing, the junctions between these cells are modified following stimulation by inflammatory mediators and growth factors, affecting barrier permeability (Brandner et al., 2006; Fishel et al., 2003; Shi et al., 2018; Tokes et al., 2013). Among these factors, VEGF, abundantly present in MVs (Arif et al., 2020), is a well‐known factor affecting BEC permeability (Collins et al., 1993; Larcher et al., 1998; Senger et al., 1983). Numerous studies have shown that EVs increase (Chatterjee, Yang, Ma, Cha, et al., 2020; Dalli et al., 2008; Hottz et al., 2013; Mantel et al., 2016; Raymond et al., 2016; Tominaga et al., 2015; Treps et al., 2016, 2017; Yang et al., 2022) or decrease (Miyazawa et al., 2019; Wang et al., 2017) endothelial barrier permeability. Our results show that MVs produced by Wmyo during wound healing can increase BEC permeability by altering intercellular junctions, leading to the formation of gaps between cells. The increase in barrier permeability induced by MVs can be linked to the presence of membrane proteins but not to VEGF, since none of the VEGF receptors were involved in the internalization of MVs. This suggests that the mechanisms underlying MV action may involve other factors besides the known VEGF receptors. Future investigations will aim to identify and characterize these additional factors, exploring possibilities such as membrane proteins or signalling pathways. For instance, our study has shown that MVs carry lymphotoxin alpha (Arif et al., 2020), a protein capable of activating endothelial cells (Fhu et al., 2014) through proteins like ICAM‐1 (Mai et al., 2013; Rao et al., 2007). These findings emphasize the complexity of MV‐induced effects on BEC permeability, extending beyond the traditional VEGF‐centric view.

In contrast, the LEC barrier is known to be more permeable than the BEC barrier due to the presence of intercellular gaps between the LECs which allow the penetration of interstitial fluid, proteins, and cells through the barrier (Leak & Burke, 1968; Potente & Mäkinen, 2017). During the early stages of wound healing, the LEC barrier shows an increase in permeability (Cromer et al., 2014; Fishel et al., 2003; Lala et al., 2018; Zhang et al., 2020), which decreases over time (Henderson et al., 2021). This increased permeability facilitates lymphatic drainage of the wound and the transport of immune cells to the lymph nodes, which may prevent complications such as oedema (Fishel et al., 2003). The action of MVs on this barrier is not known, but previous results have shown that there is an influx of EVs into the lymph, allowing EVs to be drained out of the tissue (Tessandier et al., 2020). Interestingly, our results indicate that MVs transiently reduced the permeability of the LEC barrier by reducing the extent of intercellular gap junctions, which may restrict the passive diffusion of larger molecules across the barrier.

Despite different action of EVs on endothelial barriers according endothelial cell origin, we have demonstrated that EVs can be internalized by both BEC and LEC, potentially influencing various functions that contribute to barrier permeability (Chatterjee, Yang, Ma, Cha, et al., 2020; Jakubec et al., 2020; Merino et al., 2021; Saint‐Pol et al., 2020; Wang et al., 2017; Zhao et al., 2018). To have a better understanding of MV internalization and their effect on cells, we evaluated the endocytic pathways used. Our study demonstrated that the mechanisms of MV internalization differ between BECs and LECs, with caveolae‐dependent endocytosis being the primary route in BECs and macropinocytosis playing a significant role in LECs. This difference of pathway may explain the rapid disappearance of MVs in LECs, which follow the lysosomal pathway, whereas in BECs, MVs can be detected in cells during at least 4 days (Supp. Data 4). This could also explain the transient action of MVs on LEC barrier permeability, this action decreasing after 24 h whereas the action of MVs on BEC remained constant even after 30 h. In this present investigation, we refrained from an in‐depth exploration into the precise entry pathways of MVs and their subsequent intracellular localization. This specific facet of our research is reserved for future inquiries. We deliberately maintained focus on the primary objective of this research paper, which is the evaluation of MV diffusion through endothelial barriers. By deferring the comprehensive investigation of MV internalization pathways, we aimed to uphold clarity and coherence in addressing the central theme of this study.

In the exploration of cellular internalization pathways, it is pertinent to consider the prospect of transcytosis, albeit its omission from our investigative purview due to observed inconsistencies with established transcytosis processes. As delineated in the ensuing paragraph, our investigation reveals the efficient internalization of MVs by BECs, contributing to increased permeability, predominantly via the paracellular route during a subsequent phase. Within LECs, MV diffusion occurred under conditions of natural barrier permeability, unveiling the potential involvement of macropinocytosis in MV uptake. Noteworthy, the subsequent decline in MV fluorescence within LECs, devoid of external diffusion, suggests a plausible trajectory involving lysosomal degradation, thereby casting doubt upon the feasibility of a linear transcytosis route. Although we showed that the BEC barrier was impermeable to MVs, we also demonstrated that their ability to cross it was enhanced when the barrier had been previously stimulated by VEGF or MVs (Supp. Data 5). This mechanism was reversed when LECs were used: after stimulation, the permeability of the LEC barrier, which is high in the absence of any stimulus, decreased sharply. Wmyo may therefore play a role in supporting capillary integrity via the production of factors such as VEGF or MVs, and thus fluid circulation in the wound. We therefore suggest that MVs may act on fluid circulation in injured tissues in a two‐step process. In the first step, MVs are internalized by BECs and LECs, affecting the integrity of intercellular junctions. This ‘first wave’ of MVs increases the permeability of the BEC barrier but decreases that of the LEC barrier, increasing the quantity of fluid present in the tissue. In a second step, a massive outflow of MVs from the tissue can be achieved, thanks to the high permeability of the BEC barrier and the return to normal of the LEC barrier permeability. This hypothesis has also been proposed for the blood‐brain barrier (Jarmalavičiūtė & Pivoriūnas, 2016) and suggests that this model could be helpful for establishing new, more effective protocols for the delivery of EVs. They suggested that an initial pretreatment with EVs could be followed by a ‘window’ allowing maximal permeabilization of endothelial barriers, followed by the next ‘therapeutic’ dose of EVs.

In considering the limitations of our in vitro transwell system, it is essential to recognize its valuable insight into MV transport through endothelial barriers or their influence on them. However, this model does not fully emulate the intricate conditions of in vivo wound healing (Heras et al., 2022; Ren et al., 2019; Ud‐Din & Bayat, 2017). While beneficial for specific aspects of MV transport, the use of endothelial cells in the transwell assay does not fully capture the dynamic nature of wound healing, which involves diverse cell types, growth factors and the extracellular matrix (Heras et al., 2022; Ren et al., 2019; Ud‐Din & Bayat, 2017). Although our controlled setting reveals key mechanisms, it inherently lacks the capacity to fully replicate in vivo nuances. Therefore, caution is warranted when extrapolating findings to the in vivo milieu, as emphasized by studies advocating for complementary ex vivo and in vivo models (Heras et al., 2022; Ren et al., 2019; Ud‐Din & Bayat, 2017). This understanding informs future research directions, endorsing comprehensive methodologies that bridge the experimental gap between controlled in vitro environments and the physiological complexity of wound healing in vivo.

## 5 CONCLUSION

Overall, our study demonstrates the diffusion capacity of Wmyo‐derived MVs across the endothelial barrier under physiological conditions, and their influence on its permeability. The intriguing results of our study lead to interesting hypotheses about the multiple functions of MVs in wound healing. These MVs mainly contain pro‐angiogenic and pro‐inflammatory mediators, suggesting their potential to trigger downstream effects after exiting the tissue. The endothelial barrier is known to be tightly regulated by specialized endothelial cells lining the inner surface of capillaries (Chatterjee, Yang, Ma, Wu, et al., 2020; Ramos‐Zaldívar et al., 2022). This barrier enables the selective exchange of elements between the tissue and the vascular system, while eliminating waste products (Mehta et al., 2014; Vandenbroucke et al., 2008). BECs are responsible for the distribution of blood and its components throughout the body, while LECs play a crucial role in the elimination of interstitial fluid accumulated in tissues, thus maintaining tissue homeostasis (Kaipainen & Bielenberg, 2010). Our findings demonstrate that MVs could be a crucial component of processes such as oedema in wound healing. This represents a significant advance in our understanding of the mechanisms of barrier permeability and could inspire the development of new therapies. This study opens up new avenues for future research and offers great potential for the development of more effective treatments for wound healing and related conditions.

## AUTHOR CONTRIBUTIONS

Syrine Arif and Véronique J. Moulin conceptualized the project; Syrine Arif performed the experiments. Syrine Arif wrote the original draft of the manuscript. Megan Richer helped with the characterization of endothelial cells. Sébastien Larochelle helped with the preparation of mNeonGreen myofibroblasts and endothelial cells isolation. Véronique J. Moulin supervised the research. All the authors reviewed and edited the manuscript.

## CONFLICT OF INTEREST STATEMENT

The authors report no conflict of interests.

## Supporting information

Supporting Information
