# Supplementary material for: Microvesicles derived from dermal myofibroblasts modify the integrity of the blood and lymphatic barriers using distinct endocytosis pathways
Source: J Extracell Biol. 2024 May 2;3(5):e151. doi: 10.1002/jex2.151 (PMC11080715; doi:10.1002/jex2.151)
Supplement: Supplementary file 1 — Supporting Information [file JEX2-3-e151-s001.docx]

**Supplementary data**


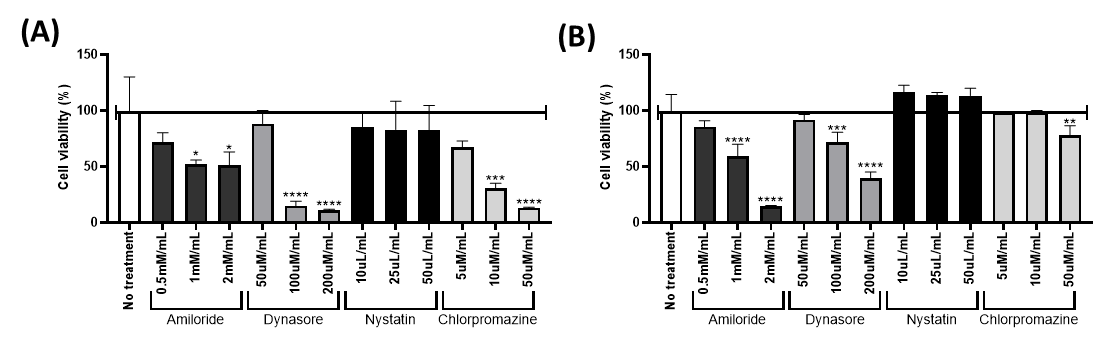


**Supp. data 1: Effect of different inhibitors dilution on BEC and LEC viability**

Figures show the effect of different inhibitors on cell viability evaluated by AlamarBlue for (**A**) BEC and (**B**) LEC. N=2, n=3. Statistical analysis is presented as mean ± SD of the relative permeability, with one-way ANOVA followed by Dunnett's test, with No treatment’' as the control. *P<0.0332, **P<0.0021, ***P<0.0002, ****P<0.0001.


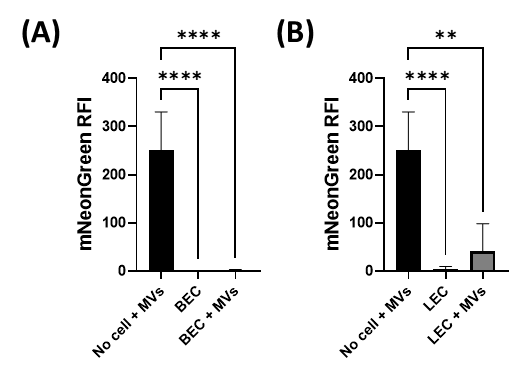


**Supp. data 2: Presence of MVs in upper transwell chamber**

A measure of the relative fluorescence intensity was used to assess the presence of MVs in the upper transwell compartment for BEC barrier **(A)** and LEC barrier **(B)**. N=3, n=3, n’=3 MVs samples isolated from 3 different Wmyo populations. Statistical test used was two-way ANOVA, Dunnett’s test (control: No cell + MVs), **P < 0.0021 ****P < 0.0001.


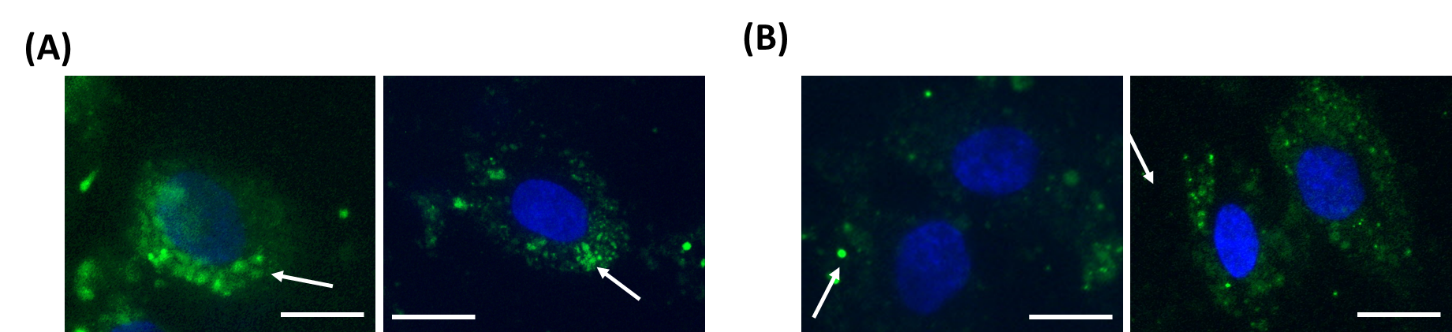


**Supp. data 3: MVs are localized in close proximity to the nucleus**

The immunofluorescence method was used to visualize the presence of mNeonGreen-labeled MVs in BEC (**A**) and LEC (**B**) after 3h stimulation. A representative image of N=1, n=3, n’=2-3 experiments is shown, where blue indicates Hoechst staining of nuclei, and green indicates the presence of mNeonGreen-labeled MVs (White arrows). The scale bar corresponds to 10 µm.


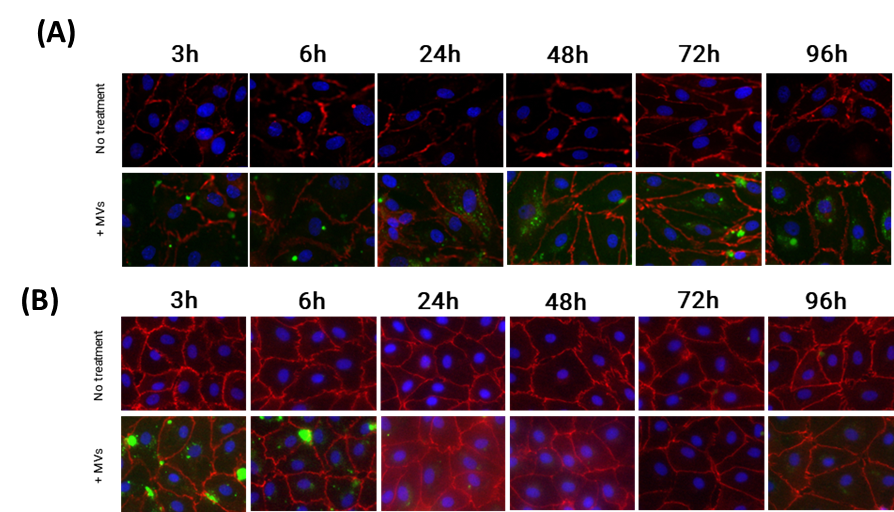


**Supp. data 4: MVs internalization over 4 days**

The immunofluorescence method was used to visualize the presence of mNeonGreen-labeled MVs in BEC (**A**) and LEC (**B**) for 4 days stimulation. A representative image of N=1-2, n=3, n’=1 experiments is shown, where red represents Ve-cadherin, blue indicates Hoechst staining of nuclei, and green indicates the presence of mNeonGreen-labeled MVs.

**
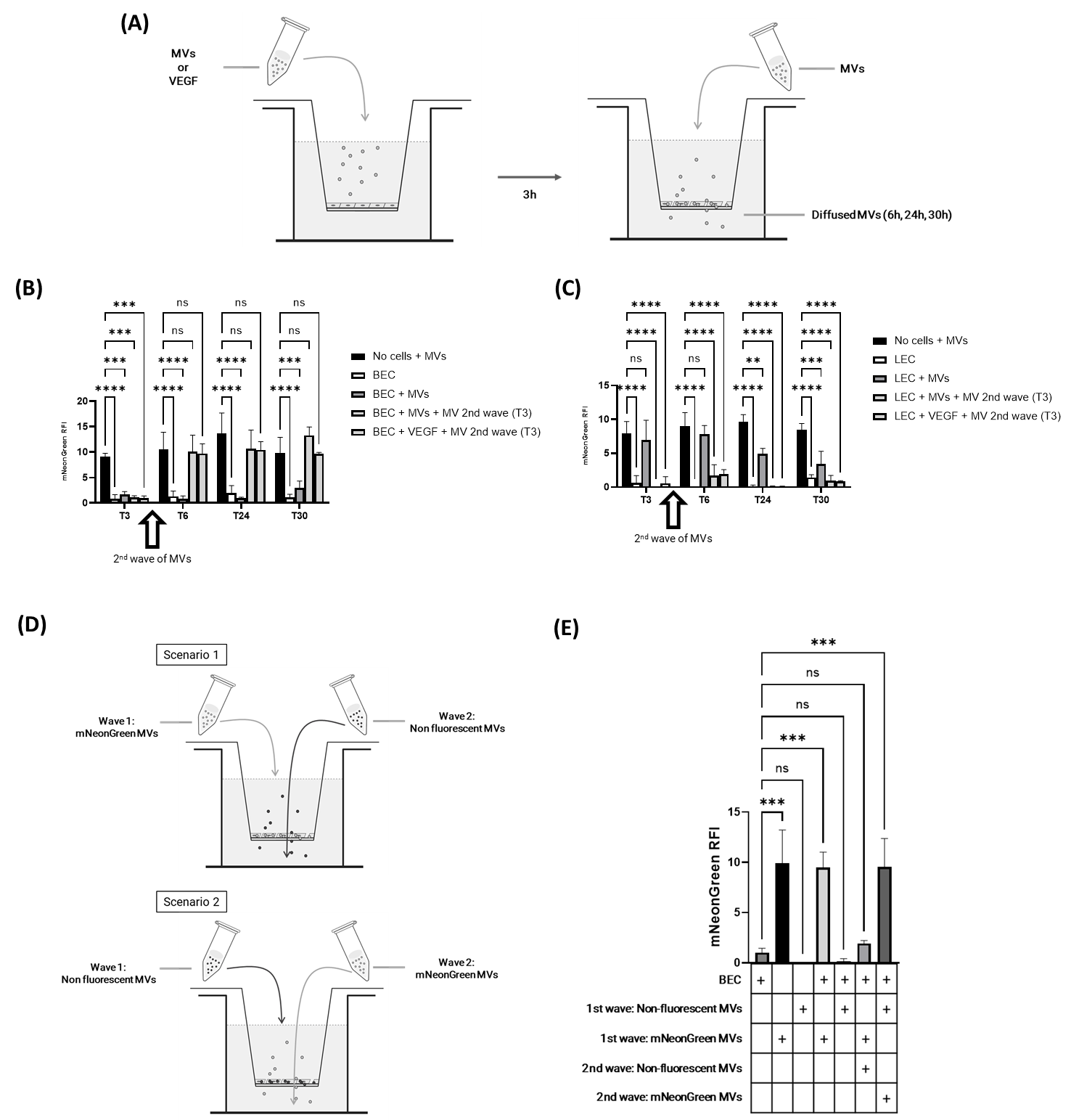
**

**Supp. Data 5: MVs diffused through a pre-permeabilized BEC barrier**

**(A)** Illustration of the proposed protocol for assessing MV diffusion across HDMEC barriers using two consecutive treatments with MVs. Evaluation of MV diffusion through the BEC **(B)** or LEC **(C) barrier** was performed by applying the first wave at the beginning and the second wave after 3 hours (indicated by the arrow).Experiments were conducted with N=2-3, n=3, n’=1-3. To evaluate the statistical significance of our results, we used two-way ANOVA with Dunnett’s test and ‘’No cells + MVs’’ as control. The results are presented as: Ns:P> 0.1234, **P< 0.0021, ***P< 0.0002, ****P<0.0001. Two experimental scenarios were used **(D)** to better explain two-wave diffusion, using fluorescent mNeonGreen MVs or non-fluorescent MVs to assess diffusion across the BEC barrier. **(E)** shows the relative fluorescence intensity of MVs in the lower compartment after 6 hours, in the presence of both waves. The experiments were conducted with N=3, n=3, n’=3. For statistical analysis, we used one-way ANOVA with Dunnett’s test and ‘’No cells + mNeonGreen MVs’’ as control. The symbols used for statistical analysis are as follows: Ns:P> 0.1234, ***P< 0.0002, ****P<0.0001.
